# Evolution of Th2 Immunity: A Rapid Repair Response to Tissue Destructive Pathogens

**DOI:** 10.1371/journal.ppat.1002003

**Published:** 2011-05-12

**Authors:** Judith E. Allen, Thomas A. Wynn

**Affiliations:** 1 Institutes of Evolution, Immunology and Infection Research, University of Edinburgh, Edinburgh, United Kingdom; 2 Immunopathogenesis Section, National Institute of Allergy and Infectious Disease, National Institutes of Health, Bethesda, Maryland, United States of America; University of California San Francisco, United States of America

## Why Did Th2 Immunity Evolve?

Throughout evolutionary history, animals have faced attack by fellow metazoans, often resulting in damage to tissues. This can take the form of a worm migrating to find its reproductive niche or eating host tissue for food, or even the bite of an insect. A pro-inflammatory oxidative-type of immunological attack, typically utilized against intracellular microbes, can in some cases kill these multicellular parasites [1], but because worms and insects cannot be contained within a single cell, the collateral tissue damage that will result from such an attack could seriously compromise host fitness. During the course of evolution, the most cost-effective approach to deal with very large foreign invaders may have been to tolerate them and quickly repair any tissue damage that compromised fitness [2], [3]. In this scenario, Th1 immunity characterized by IFN-γ production evolved to control our innate anti-microbial pathways, while the host defense system that evolved to cope with metazoan parasites was the innate tissue repair process, now controlled by Th2 cells. Th2 cells subsequently evolved additional mechanisms to contain or even expel the offending element and produce cytokines such as IL-4, IL-5, IL-10, and IL-13 that promote alternative macrophage activation, eosinophil maturation and recruitment, and IgE production, to name just a few [4]. Many of these Th2 processes promote the “walling off” of large bodies through granuloma formation and matrix deposition, which would quite naturally follow from mechanisms evolved to close open wounds.

**Figure 1:**
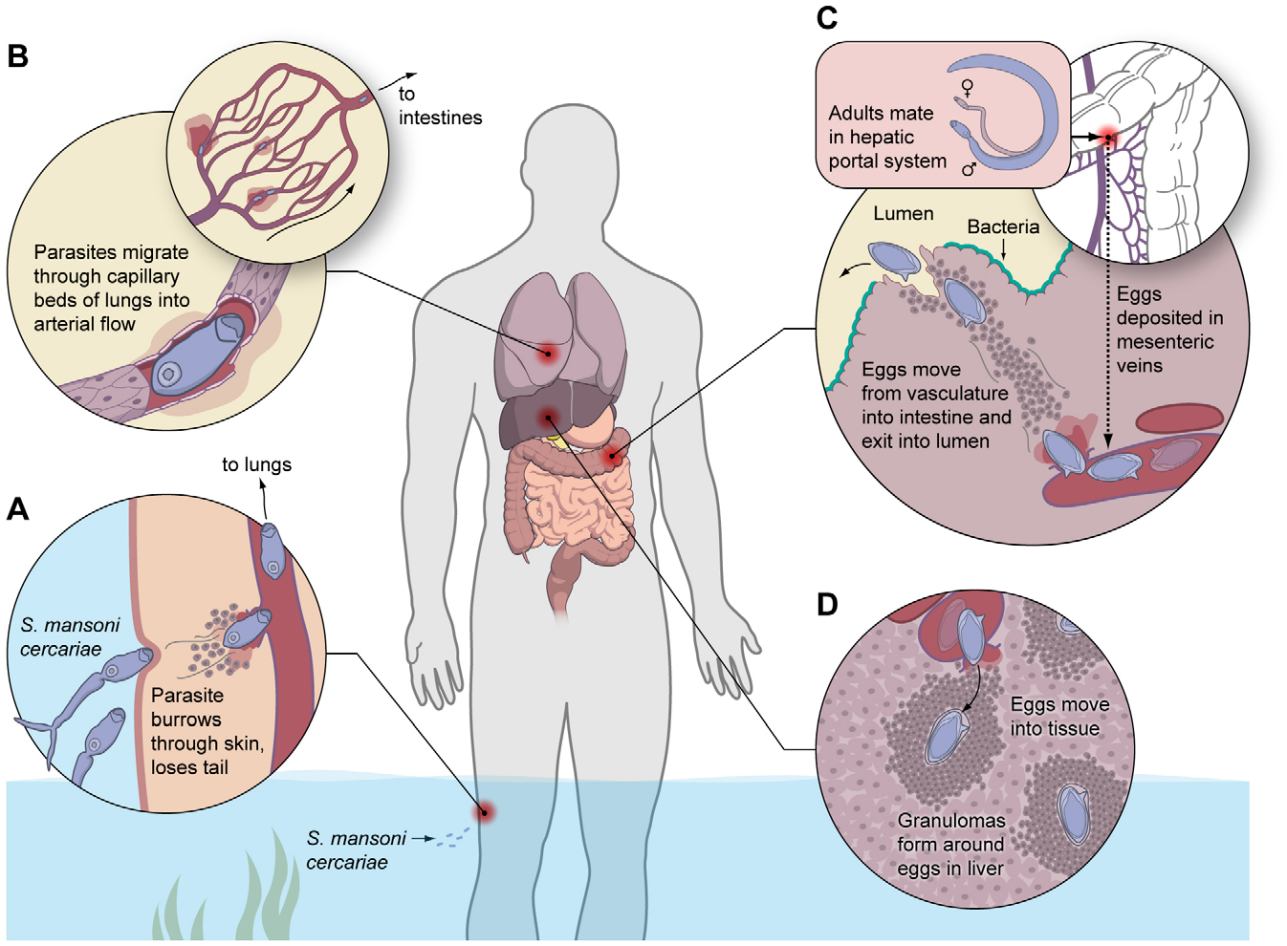
Helminths induce extensive tissue damage, providing evolutionary pressure for an adaptive Th2-mediated wound healing response. In this example, a human infected with the helminth parasite *S. mansoni* is faced with constant tissue damage as the parasite completes its life cycle. (A) Infectious cercariae are released from the intermediate snail host and are attracted to lipids found on human skin. Once attached to the skin, they often enter through hair follicles where they secrete proteases, degrade basement membranes, and ultimately gain access to the vasculature. (B) Immature schistosomula are then swept up in the heart and lodge in the lungs, where they must cross capillary beds to enter the arterial flow. (C) Eventually, adult parasites find their way to the mesenteric veins, where they mate and begin laying eggs. Many of the eggs migrate from the vasculature, enter the wall of the intestine and literally burrow through until they reach the lumen and are excreted in the feces. (D) A subset of eggs is swept by the blood flow into the liver where they are trapped in the small sinusoidal vessels, inducing a vigorous granulomatous response. Thus, at nearly all stages of the parasite's life cycle, it is inducing significant tissue damage and hemorrhaging in the definitive host. It is imperative that “holes” in important barriers are repaired quickly; otherwise, bacteria would quickly invade and take over.

Evolutionary hypotheses are difficult to prove, but murine studies of helminth infection provide “modern” evidence that tissue repair orchestrated by Th2 cells is a primary host defense against metazoa. As illustrated in Figure 1 for *Schistosoma mansoni*, metazoan invaders literally tear through important barriers, often inducing micro-hemorrhages and tissue damage in multiple organs as they complete their life cycle (Figure 1). Strikingly, *S. mansoni* infection of IL-4Rα-deficient animals that lack most Th2 effector responses results in lethal sepsis once eggs produced in the mesenteric blood vessels cross the intestinal wall [5]. This suggests that IL-4Rα-mediated pathways are critically needed to maintain gut integrity and prevent leakage of luminal dwelling bacteria into the blood. A similar scenario plays out during infections with many gut nematodes, with broad-spectrum antibiotics providing at least partial protection from sepsis when IL-4Rα-driven barrier immunity is impaired [6].

The cardinal features of adaptive immunity are memory and antigen-specificity. Since Th2 cells are part of the adaptive immune system, this raises the question of why we need to “remember” to repair the wounds that are induced by specific parasites. A hookworm causes bleeding as it migrates through the lung and then penetrates the gut wall to feed. A parasite-specific memory Th2 cell might accelerate wound closure, significantly reducing detrimental effects on secondary exposures. Indeed, adaptive immunity and memory may be equally important for tolerance mechanisms that minimize host damage as they are for resistance to the pathogen itself [3], [7]. To date, no experiments have directly addressed whether wounds repair faster on a secondary encounter with the same injuring agent. However, there is evidence to suggest that hemorrhaging is reduced on secondary infection with lung-migrating nematodes (Graham LeGros, personal communication).

Helminths, the best-described inducers of Th2 cytokines, include parasites from animal phyla that diverged over a billion years ago, and increasing evidence suggests that insect bites are also Th2-inducers [8], [9]. Thus, it appears that we are hard-wired to mount Th2 responses to an attack by any metazoan pathogen. Tissue destruction is a common feature of these parasites, and we are proposing that Th2 immunity evolved as an adaptive tissue repair mechanism that quickly heals the wounds they inflict. These evolutionary principles, if true, must apply beyond mammals. Infection of Atlantic salmon with sea lice causes gross skin lesions that must be rapidly healed, as any break can result in osmotic shock in the aqueous environment. Activated Th2 cells migrate to the site of attachment and may mediate essential repair of the lesion but also expulsion of the ectoparasite [10]. Importantly, an anti-wounding response is not unique to vertebrates, but one of the fitness advantages provided by the adaptive immune system may have been the ability to accelerate this response as needed, to mediate parasite-specific tolerance [3].

## What Evidence Supports the Theory of Th2-Mediated Repair?

During an ideal wound repair response, the damaged tissues are returned to their original architecture. However, the process of mending damaged tissues takes considerable time, so the body responds quickly during the early phases of parasite invasion by sealing the wounds with granulation tissue, which essentially provides a “quick fix” and prevents neighboring bacteria from invading. Granulation tissue is the fibrous connective tissue that replaces a fibrin clot in healing wounds. It typically forms at the border of a wound and is able to fill wounds of almost any size. Initially, it consists of a network of Type III collagen, a weaker form of the structural protein that is produced rapidly by activated fibroblasts. This is later replaced by the stronger, long-stranded Type I collagen. Importantly, numerous studies have suggested that the synthesis of both Col I and Col III during helminth infection is highly dependent on the Th2 cytokines IL-4 and IL-13 [11].

In fact, many of the proteins produced in response to IL-4 and IL-13 are associated with injury, and several, including arginase, MMP12, and TREM-2, have well-known roles in tissue repair. Evidence that Th2-dependent pathways are a normal part of tissue repair comes from a study in which a surgical incision in the peritoneal wall induces rapid elevation of Th2-associated proteins, arginase, RELMα, and YM1, but only in mice with intact IL-4Rα signalling [12]. More recently, these same proteins were demonstrated in wound tissue in the first 5 days of a punch biopsy wound model [13]. Naturally, these IL-4/13-dependent proteins are also elevated during helminth infection, where the parasite is presumed to be the Th2 stimulus [14]. During infection with *Nippostrongylus brasiliensis*, Th2-induced proteins are particularly elevated during migration of nematode larvae through the lung [15], a process that is highly damaging and leads to hemorrhaging that is sufficient to cause anemia [16]. Although CD4^+^ Th2 cells are needed for sustained and high-level production of these injury-associated proteins [12], arginase, Ym1, and RELMα and are still produced in an IL-4Rα-dependent manner in RAG-/- mice, emphasizing the innate nature of the response [12], [15].

Although there is good evidence that Th2 cytokines are associated with injury, their actual contribution to repair is not clear. A study by Seno et al. provides important insight [17]. Using a colonic punch biopsy model, they demonstrated that IL-4/IL-13 blockade or deficiency in IL-4Rα signaling leads to a delay in wound repair. Similarly, using a skin biopsy model, Sabine Eming and colleagues have demonstrated that IL-4Rα-deficient mice also show significantly delayed repair (personal communication). Thus, despite very different healing processes in the colon and skin [17], the rate and quality of repair in both are affected by IL-4 receptor signaling. Indeed, delayed repair is a feature of several mouse strains with deficiencies in Th2-induced proteins [17], [18].

## What Are the Consequences of Th2-Mediated Rapid Repair?

Probably the most extensive evidence for the involvement of Th2 cytokines in tissue repair comes from studies demonstrating that IL-13 is a potently pro-fibrotic cytokine [11]. Thus, in order to maintain tissue integrity, Th2 cytokines may accelerate repair but at the cost of scar tissue. A key cellular target of the IL-13 response is the macrophage, which, when activated by Th2-type cytokines (M2 macrophage), has been shown to control the development of fibrosis [19]. In the early stages of repair, macrophages produce a variety of factors that recruit and activate fibroblasts, while in the later stages, they are involved in wound resolution by debriding the wound, inducing apoptosis of myofibroblasts, and producing regulatory factors like Arg1, which can suppress T cell proliferation [19]. Recently, Lucas and colleagues [13] demonstrated that macrophages recruited during the early inflammatory stages of a sterile skin wound expressed Th2 activation markers and that macrophage depletion in the first 5 days significantly delayed the rate of repair but also resulted in less scar tissue. This is consistent with studies in which mice that lack macrophages exhibit no scar tissue [20]. The data suggest that Th2-activated M2 macrophages ensure rapid wound closure, while at the same time regulating matrix turnover and wound resolution and thus the subsequent process of scarring [19].

## Why Is Th2 Immunity “Anti-Inflammatory”?

The wound-healing hypothesis provides a framework to consider many aspects of T helper cell biology, including understanding why naive T cells commit to a particular lineage at the expense of another. Evolutionary models have helped explain the Th17/Treg counterbalance [21], but the origins of the Th1/Th2 divide are not obvious. The requirement for counter-regulation becomes apparent when one considers the roles of inflammatory responses in wound repair. An injury response typically begins with a classical inflammatory response, composed of neutrophils and IFN-γ/TLR-activated M1 macrophages that control microbial contamination. However, the M1 activation pathway is only essential to the repair process if microbes are present [17], [22] and thus functions primarily to control infection and not mediate repair. Indeed, efficient wound closure and full repair cannot occur until that inflammatory response has been shut down [23], [24]. Thus, the anti-inflammatory nature of “regulatory” Th2 responses makes evolutionary sense if the responses to metazoans are primarily tissue reparative rather than anti-microbial. On exposure to helminths, the host would avoid or quickly shut down an ineffective and damaging Th1-type response in favor of a mechanism that would “rapidly and adaptively” heal the host and thus allow it to tolerate the presence of a persistent pathogen [3], [7], [25]. Consistent with this, a mixed anti-inflammatory/wound healing function is typical of many Th2-activated macrophage products. TGF-β is the best-known example, as it can suppress pro-inflammatory responses while at the same time serving as a potent pro-fibrotic mediator. Similarly, TREM-2 and 12/15-lipoxygenase, both induced by IL-4, are well-known anti-inflammatory mediators; however, both appear to accelerate wound repair [17], [26], [27]. Indeed, one mechanism by which these proteins may accelerate the repair process is to rapidly shut down the early inflammatory response to injury. This dual function would also be consistent with the need to sequester parasites by wrapping them in collagen, much as flies wrap parasitoids in melanin.

## How Can This Evolutionary Model Help Us Today?

We mount Th2 responses to helminths, environmental allergens, and insect bites. A major outstanding question is the nature of the signals and receptors that trigger these responses [3], [25]. A common feature of these insults is the ability to damage tissue. In particular, proteases have been highlighted for their capacity to induce Th2 immunity [28]. Alum, an established Th2 adjuvant, acts by triggering uric acid release, a signal of cell damage. Recently, several groups have identified a new innate immune cell that produces IL-5 and IL-13 [29]. A critical player in inducing the release of these Th2 cytokines is IL-33. IL-33 is released by endothelial, epithelial, fibroblast, and adipose cells only when they die and thus may be a critical player in inducing a “Th2 injury” response. Similar roles may also be played by TSLP and IL-25, which have also been proposed as important early inducers of Th2 responses [30].

We are not arguing that all aspects of Th2 immunity now extant are involved in healing wounds. The threats metazoan pathogens pose are distinct from smaller microbes and require an array of distinct responses, a dichotomy observed in all multicellular hosts, even plants. As the innate repair machinery evolved into a full blown Th2-adaptive response, repair processes would have become associated with other features of defense that increase the fitness of its host in the face of large metazoan parasites or the toxins they release. Thus, Th2 cytokines mediate rapid repair while also minimizing the number of incoming parasites via IgE or flushing out intestinal parasites via alterations to gut physiology and excess mucus production. Over time these pathways have become increasingly specialized, providing further rationale for Th subset plasticity and subdivision into discrete cytokine-producing cells such as follicular helper and Th9 cells [31]. Nonetheless, an understanding that Th2 immunity in vertebrates evolved as a means to rapidly repair tissue damage caused by metazoan invaders rather than just to control parasite numbers may help in the development of strategies to appropriately target helminth infections as well as diseases caused by overzealous repair.

## References

[1] Thomas GR, McCrossan M, Selkirk ME (1997). Cytostatic and cytotoxic effects of activated macrophages and nitric oxide donors on *Brugia malayi*.. Infection and Immunity.

[2] Graham AL, Allen JE, Read AF (2005). Evolutionary causes and consequences of immunopathology.. Ann Rev Ecol Evol Syst.

[3] Schneider DS, Ayres JS (2008). Two ways to survive infection: what resistance and tolerance can teach us about treating infectious diseases.. Nat Rev Immunol.

[4] Diaz A, Allen JE (2007). Mapping immune response profiles: The emerging scenario from helminth immunology.. Eur J Immunol.

[5] Herbert DR, Holscher C, Mohrs M, Arendse B, Schwegmann A (2004). Alternative macrophage activation is essential for survival during schistosomiasis and downmodulates T helper 1 responses and immunopathology.. Immunity.

[6] Schopf LR, Hoffmann KF, Cheever AW, Urban JF, Wynn TA (2002). IL-10 is critical for host resistance and survival during gastrointestinal helminth infection.. J Immunol.

[7] Raberg L, Graham AL, Read AF (2009). Decomposing health: tolerance and resistance to parasites in animals.. Philos Trans R Soc Lond B Biol Sci.

[8] Brossard M, Wikel SK (2004). Tick immunobiology.. Parasitology.

[9] Boppana VD, Thangamani S, Adler AJ, Wikel SK (2009). SAAG-4 is a novel mosquito salivary protein that programmes host CD4 T cells to express IL-4.. Parasite Immunol.

[10] Skugor S, Glover KA, Nilsen F, Krasnov A (2008). Local and systemic gene expression responses of Atlantic salmon (Salmo salar L.) to infection with the salmon louse (Lepeophtheirus salmonis).. BMC Genomics.

[11] Wynn TA (2004). Fibrotic disease and the T(H)1/T(H)2 paradigm.. Nat Rev Immunol.

[12] Loke P, Gallagher I, Nair MG, Zang X, Brombacher F (2007). Alternative activation is an innate response to injury that requires CD4+ T cells to be sustained during chronic infection.. J Immunol.

[13] Lucas T, Waisman A, Ranjan R, Roes J, Krieg T (2010). Differential roles of macrophages in diverse phases of skin repair.. J Immunol.

[14] Nair MG, Gallagher I, Taylor M, Loke P, Coulson PS (2005). Chitinase and Fizz family members are a generalized feature of nematode infection with selective upregulation of Ym1 and Fizz1 by antigen presenting cells.. Infect Immun.

[15] Reece JJ, Siracusa MC, Scott AL (2006). Innate immune responses to lung-stage helminth infection induce alternatively activated alveolar macrophages.. Infect Immun.

[16] Hoeve MA, Mylonas KJ, Fairlie-Clarke KJ, Mahajan SM, Allen JE (2009). Plasmodium chabaudi limits early Nippostrongylus brasiliensis-induced pulmonary immune activation and Th2 polarization in co-infected mice.. BMC Immunology.

[17] Seno H, Miyoshi H, Brown SL, Geske MJ, Colonna M (2009). Efficient colonic mucosal wound repair requires Trem2 signaling.. Proc Natl Acad Sci U S A.

[18] Gronert K, Maheshwari N, Khan N, Hassan IR, Dunn M (2005). A role for the mouse 12/15-lipoxygenase pathway in promoting epithelial wound healing and host defense.. J Biol Chem.

[19] Wynn TA, Barron L (2010). Macrophages: master regulators of inflammation and fibrosis.. Semin Liver Dis.

[20] Martin P, D'Souza D, Martin J, Grose R, Cooper L (2003). Wound healing in the PU.1 null mouse--tissue repair is not dependent on inflammatory cells.. Curr Biol.

[21] Weaver CT, Hatton RD (2009). Interplay between the TH17 and TReg cell lineages: a (co-)evolutionary perspective.. Nat Rev Immunol.

[22] Mahoney E, Reichner J, Bostom LR, Mastrofrancesco B, Henry W (2002). Bacterial colonization and the expression of inducible nitric oxide synthase in murine wounds.. Am J Pathol.

[23] Ashcroft GS, Mills SJ, Lei K, Gibbons L, Jeong MJ (2003). Estrogen modulates cutaneous wound healing by downregulating macrophage migration inhibitory factor.. J Clin Invest.

[24] Eming SA, Krieg T, Davidson JM (2007). Inflammation in wound repair: molecular and cellular mechanisms.. J Invest Dermatol.

[25] Medzhitov R (2010). Innate immunity: quo vadis?. Nat Immunol.

[26] Kuhn H, O'Donnell VB (2006). Inflammation and immune regulation by 12/15-lipoxygenases.. Prog Lipid Res.

[27] Turnbull IR, Gilfillan S, Cella M, Aoshi T, Miller M (2006). Cutting edge: TREM-2 attenuates macrophage activation.. J Immunol.

[28] Donnelly S, Dalton JP, Loukas A (2006). Proteases in helminth- and allergen- induced inflammatory responses.. Chem Immunol Allergy.

[29] Strober W (2010). Immunology: The expanding T(H)2 universe.. Nature.

[30] Saenz SA, Taylor BC, Artis D (2008). Welcome to the neighborhood: epithelial cell-derived cytokines license innate and adaptive immune responses at mucosal sites.. Immunol Rev.

[31] Locksley RM (2009). Nine lives: plasticity among T helper cell subsets.. J Exp Med.

